# More than the worksite cafeteria: the workplace food environment of small and medium-sized enterprises in the Netherlands

**DOI:** 10.1017/S1368980024000946

**Published:** 2024-04-29

**Authors:** Lisanne Geboers, Emely de Vet, Frédérique C Rongen, Maartje P Poelman

**Affiliations:** Chair Group Consumption and Healthy Lifestyles, Department of Social Sciences, Wageningen University & Research, Wageningen, the Netherlands

**Keywords:** Food environment, Small and medium-sized enterprises, Number of employees, Work location, Worksite cafeteria

## Abstract

**Objective:**

To characterise the food environment of Dutch small and medium-sized enterprises (SMEs), encompassing physical, sociocultural, economic and policy features and to explore variations within SMEs according to company characteristics (number of employees, location of work and presence of worksite cafeteria).

**Design:**

Online cross-sectional survey study of a representative Dutch SME sample by a panel agency.

**Setting:**

Dutch SMEs.

**Participants:**

Three hundred and fifteen employees of Dutch SMEs responsible for food and drink in their company.

**Results:**

Most SMEs did not have a worksite cafeteria, no provision of fruits or vegetables, and did not offer discounts on food or drinks. The food environment of these SMEs varied significantly based on company characteristics. For example, SMEs with a worksite cafeteria were significantly more likely to have fruits (OR = 8·76, 95 % CI (4·50, 17·06)), vegetables (OR = 10·29, 95 % CI (5·49, 19·31)) and company food policies (OR = 5·04, 95 % CI (2·08, 12·20)) than SMEs without. Additionally, SMEs with ≥ 50 employees were more likely to have fruits (OR = 2·39, 95 % CI (1·42, 4·03)), vegetables (OR = 1·89, 95 % CI (1·04, 3·46)) and company food policies (OR = 2·82, 95 % CI (1·09, 7·29) than SMEs with < 50 employees. Moreover, having a worksite cafeteria (B = 0·23, 95 % CI (0·08, 0·38)) and employees working mostly on-site (B = 0·14, 95 % CI (0·01, 0·28)) were associated with stronger social norms of healthy and sustainable eating at work compared to SMEs without a worksite cafeteria and working mostly off-site.

**Conclusions:**

In SMEs, an overall comprehensive picture of the food environment points to its limited active encouragement of healthy food choices, particularly so in small SMEs without a worksite cafeteria. Company characteristics strongly influence SME food environments and should be considered when developing interventions improving SME workplace food environments.

Unhealthy and unsustainable diets are a major public and planetary health concern, contributing to the burden of diet-related non-communicable diseases and climate change^(1)^. Promoting healthy and sustainable diets is therefore a key public health priority^(2,3)^. While a myriad of factors steer food consumption, unhealthy and unsustainable diets are largely driven by current food environments, such as the availability and accessibility of food and drinks in our living environment (e.g. via supermarkets, restaurants and cafeterias). Currently, the food environment predominantly encourages poor food choices^(4,5)^. Given the substantial time people spend at work, with full-time employees dedicating a median of 40·5 h per week at work in OECD countries^(6)^, the workplace food environment may affect dietary choices. Employees consume approximately a third of their daily energy intake at the workplace^(7)^, and in many European countries, lunch is often consumed within the workplace premises^(8)^. Similarly, in the Netherlands, 77 % of employees spend their lunch breaks at work, with employees opting to spend their lunch at their desks (21 %), in the office break area (37 %) or at the worksite cafeteria (19 %)^(8)^. Hence, the workplace food environment holds significant potential as a leverage point to foster healthy diets.

However, a gap arises because there is a limited understanding of the characteristics of all dimensions of the workplace food environment, with most insights mainly capturing the physical food environment^(9–12)^. In general, the food environment can be defined as the collective physical (availability, quality and advertisements), economic (costs), policy (rules) and sociocultural (norms and beliefs) surroundings, opportunities, and conditions that influence food and beverage consumption^(13)^. At the organisational level, the food environment encompasses the institutional level (e.g. eating spaces made available), internal level of the eating spaces (e.g. prices, promotion), the surroundings (e.g. outside establishments that sell food) and the decisional level (e.g. policies and institutional culture)^(14)^. For example, the workplace food environment of four metropolitan bus garages in the USA showed that all garages had vending machines, microwaves and refrigerators, with only 15 % of the vending machine foods meeting the criteria for healthful choices^(12)^. Furthermore, a recent systematic review revealed that, beyond the physical food environment, other factors also had an impact on eating behaviours^(7)^. To illustrate, factors related to job roles (e.g. work stress), cost of food and social dynamics (e.g. social norms) at work were linked to food consumption^(7)^. The social dynamics during lunch breaks not only stimulate stronger connections with colleagues but in turn also exert both positive and negative influences on healthy eating behaviour at the workplace. The lunch break’s culture, encompassing factors like location and the source of the meal, further contributes to these influences^(8)^. Furthermore, food policies at work prohibiting the consumption of all fast foods, sweets, and sugary soft drinks and providing free fruit have been shown to be effective in limiting the consumption of foods and drinks high in sugar and increasing fruit intake at work^(15,16)^. Additionally, discounts specifically for healthy foods have been shown to increase healthy food sales in worksite cafeterias^(17)^. These findings can be linked to policy, economic and sociocultural facets of the workplace food environment, indicating that the impact of the workplace food environment goes beyond mere physical factors such as the availability of healthy foods. Despite the importance and relevance of these insights, we currently lack a comprehensive picture of workplace food environments that encompasses all domains, including physical, sociocultural, economic and policy aspects. Our study aims to fill this gap by providing comprehensive insights across all facets of the food environment.

A second gap arises from our limited understanding of how food environments are influenced in smaller companies *without* worksite cafeterias, mainly due to the prevailing focus on workplace food environments in large companies *with* worksite cafeterias^(17–22)^. However, 99 % of companies in the European Union (EU) are small and medium-sized enterprises (SMEs), with a maximum of 250 employees^(23)^. These SMEs collectively employ approximately 100 million people within the EU^(24)^ and 4 million within the Netherlands^(25)^. Therefore, in the present study, we focus on SMEs as an understudied area, yet an important setting to study employees’ food environment and food consumption. These SMEs frequently lack worksite cafeterias due to the lack of financial viability^(26)^, which may notably impact the physical and economic facets of the food environment within these SMEs. In addition, irrespective of the presence of a worksite cafeteria, prior research has shown that smaller companies are generally less likely to offer workplace programmes promoting employees’ health than larger companies^(27–29)^. These findings suggest differences in the policy food environment, which could potentially extend to practices related to other features of the food environment. Given the differences between SMEs in the presence of a worksite cafeteria and the number of employees, our study examines worksite food environments while acknowledging these distinct characteristics.

Thirdly, SME workplaces vary considerably according to the nature of the work and therefore the type of workplaces also differ greatly among SMEs^(30)^. Employees working on-site, particularly in office settings, may have easier access to conveniently located food options, including worksite cafeterias, in-house facilities or nearby food services. On the other hand, employees working off-site, such as those in transportation or remote locations, often have limited access to healthy food options and may be exposed more often to unhealthy foods (e.g. on-the-go food outlets)^(31)^. Therefore, we hypothesise that different types of workplaces (working on-site (e.g. IT sector) *v*. off-site (e.g. transport sector)) can substantially shape exposure to food environments and that employees working off-site may have less access to healthy food than those working on-site^(31)^. The food environment of on-site and off-site employees may be further influenced by health policies at work. As indicated by the findings of Seward and colleagues^(32)^, employees working off-site generally have access to fewer health promotion programmes compared to those working on-site. Drawing upon these findings, it could be hypothesised that on-site work environments may prioritise employee health and wellbeing differently, potentially resulting in the adoption of policies and initiatives aimed at encouraging healthier food options at the workplace.

Given these considerations, this study aims to characterise the food environment of SMEs in the Netherlands in a comprehensive manner encompassing the physical, economic, policy and sociocultural aspects and to examine how the food environment varies within SMEs according to (1) the number of employees, (2) location of work and (3) presence of a worksite cafeteria. These insights are crucial, as they contribute to the advancement of knowledge regarding how workplace factors interact with the food environment. Additionally, this understanding can support employers, policymakers and health professionals in formulating interventions that align with the unique circumstances of different types of SMEs, ultimately promoting better employee health and well-being.

## Methods

### Study design

A cross-sectional survey was conducted amongst an online panel, recruited, by panel agency Flycatcher Internet Research B.V. The panel comprises individually applied Dutch participants, collectively representing the Dutch population. During the application process for joining the online panel, panel members submitted background details including the company size or responsibilities at work (if applicable).

### Procedure and participants

To gain insight into the food environments of SMEs, we recruited employees or employers of SMEs nationwide that, during the application process for the panel indicated to be (co)responsible for the ‘food and drink’ in their company. Eligible respondents were invited by email to participate in the survey. The inclusion criterion was that respondents had to be working in an SME (i.e. an organisation with 2–250 employees). Respondents working in the catering or healthcare industry were excluded due to their food environments being designed not only for employees but also for visitors and patients. Respondents were invited between December 2021 and March 2022, reflecting a situation during the COVID-19 pandemic.

An initial number of 2200 invitations for the survey were sent to employees or employers who were responsible for the food and drink. A total of 469 respondents started the survey. Of the 469 participants, the panel agency excluded respondents who did not consent to using their data (*n* 6, 1·3 %), respondents who were not eligible due to not complying with the selection criteria stated in the survey (*n* 119, 25·4 %), participants with incomplete surveys (*n* 22, 4·7 %) or a poor response quality resulting from straight lining, responding too quickly and incoherent answers to open questions (*n* 7, 1·5 %) were excluded from the analyses. A final sample of 315 respondents (14·3 % of the initial invited) was included in the analyses. This final included sample is in close alignment with the TNO Employers Survey of 2021, showing comparable sector distributions with minor variations^(33)^. While our sample’s company size differs from the national SME landscape^(34)^, intentionally incorporating diverse SME types enhances the study’s representativeness.

### Measures

The survey consisted of questions about participant and company characteristics as well as measures about the physical, sociocultural, economic and policy food environment of the workplace. These measurements were aligned with the Analysis Grid for Environments Linked to Obesity (ANGELO) framework’s definition of the food environment^(4)^. The exclusion of the more narrow organisational food environment definition to construct the measures was deliberate, as this presupposes the provision of food at the workplace, a concept not always suitable for SMEs (that do not always offer food at work)^(35,36)^. Furthermore, the survey was pretested by two employees of Flycatcher Internet Research B.V.

#### Participant characteristics

Sex, age and job position (based on a previous employer study^(37)^) were assessed. Additionally, the sector in which participants worked was assessed from a predefined list (e.g. construction and industry^(37)^).

#### Implications of COVID-19 on food and drink offer and eating practices

The survey included four items to identify the impact of the COVID-19 pandemic on the SME food environment. First, it was inquired if the offer of food and/or drinks at the workplace had changed due to the COVID-19 pandemic. Second, if respondents indicated that the offer of food and/or drinks at the workplace had indeed changed due to COVID-19, they were asked what had changed with an open-ended question. Third, it was asked if eating practices of employees had changed as a result of the COVID-19 pandemic. Lastly, if respondents indicated that eating practices of employees had indeed changed due to COVID-19, they were asked what had changed with an open-ended question.

#### Company characteristics

Company characteristics were assessed by three items. First, the total number of employees (2–250) was defined, and answers were recoded into two categories of fifty and more (≥ 50) or less than fifty (< 50) employees. The cut-off of fifty employees was used because small enterprises are defined as having less than fifty employees, and medium-sized enterprises have 50–250 employees^(23)^. Second, to determine the primary location of work there were three response options (‘all or most employees work on-site’, ‘half the employees work on-site’ and ‘all or most employees do not work on-site’)

These options were recoded into two categories: ‘most employees work on-site’ and ‘most employees work off-site (including both half the employees work on-site and all or most employees do not work on-site). On-site work was defined in the survey as working in settings such as offices or stores, while off-site work was specified as activities such as working at client’s premises or engaging in transportation-related tasks. Finally, the presence of a worksite cafeteria was identified (yes/no). Respondents (*n* 67) who indicated having a ‘worksite cafeteria’ but also indicated that no food and drinks were available for sale at work were recoded into having a ‘canteen’ (common break room) only.

#### Physical food environment influences

Three measures were used to assess the physical food environment. First, the presence of amenities at the worksite was assessed from a predefined list containing seven items (worksite cafeteria (mentioned above), catered lunch, soft drink vending machine, coffee and tea vending machine, snack vending machine, water tap and kitchen). When inquiring about snacks, the reference was unhealthy snacks (as per the Dutch translation). Hereafter, the term ‘snacks’ will be thus used to denote unhealthy snacks. Second, based on the NEMS-P survey^(38)^, respondents had to indicate whether eleven predefined kitchen appliances were available at work for employees, such as microwaves or refrigerators (see Appendix 1 for the full list). Lastly, based on the ‘Guidelines for the food environment’ of the Netherlands Nutrition Centre, the availability of certain food and drinks at the workplace was assessed, including fresh fruits, vegetables and/or salads, and ‘other’ products/meals^(39)^. If respondents indicated that other products/meals were available, they were asked about the availability of other foods and drinks from a predefined list (e.g. sandwiches, sweet snacks and sugary soft drinks) in more detail (see Appendix 2 for the full list).

#### Sociocultural food environment influences

Four variables were included to determine the sociocultural food environment. Based on Corvo and colleagues^(8)^, two questions were included regarding lunch break habits. First, respondents were presented with a predefined list and were asked to indicate the most common ways their colleagues typically spent their lunch breaks during working hours (in a communal break room or canteen/behind a desk/walking/at an external eating facility/at home/on-the-go) and the latter four options were categorised into ‘out-of-the-office’. Second, participants were required to select, from a predefined list, the primary sources from which their colleagues typically brought lunch. The options included bringing it from home, buying it from a local food provider such as a bakery or supermarket, buying it from the worksite cafeteria or canteen, groceries purchased by a colleague, or ordering lunch for delivery. The answer options from a local provider, groceries bought by a colleague and lunch ordered for delivery were recoded during data analysis into one answer option called ‘purchased elsewhere’. Third, a variable was included measuring social norms of healthy and sustainable eating at work. Respondents indicated on a five-point Likert scale (e.g. 1 = totally disagree to 5 = totally agree) to what extent they agreed on the following six statements; ‘There is a healthy eating culture at work.’; ‘There is a sustainable eating culture at work.’; ‘In general, colleagues have a positive attitude towards healthy food.’; ‘In general, colleagues have a positive attitude towards sustainable eating.’; ‘My colleagues eat snacks when they are at work.’; and ‘My colleagues eat vegetables and/or fruit when they are at work.’. A higher rating (1–5) indicated healthier and more sustainable choices, leading to the reverse-coding of the statement about snacking. The statements were based on Rongen et al.^(40)^ and were preceded by an explanation of what a healthy and sustainable diet constituted ^(41)^(see Appendix 3 for the definitions). A mean score for the six items was calculated to represent ‘social norms of healthy and sustainable eating at work’. Internal consistency was sufficient (Cronbach’s α = 0·77).

Company food policies shape food environments, impacting resources and incentives for healthy eating^(42)^. Whether these policies exist depends on underlying values/beliefs within the company, which can range from supporting employee’s health as a responsibility of the employer or the employee’s own responsibility^(43)^. The value assigned to this, including companies’ responsibility to create healthy and sustainable food environments, is part of its sociocultural food environment^(4)^.

Finally, inspired by McCleary et al.^(44)^, a variable was included to measure the extent to which respondents agreed that employers were responsible for facilitating and reimbursing healthy and sustainable food environments and stimulating the general health of their employees at the workplace. Respondents indicated on a five-point Likert scale (e.g. 1 = totally disagree to 5 = totally agree) to what extent they agreed on the following five statements; ‘Employers must play an active role in facilitating a healthy and sustainable food supply at work’; ‘Employers must play an active role in stimulating healthy and sustainable eating behaviour of their employees at work.’; ‘Employers must play an active role in stimulating the general health of their employees’; ‘The costs of facilitating a healthy and sustainable food supply among employees must be reimbursed by the employer’; and ‘The costs of stimulating healthy and sustainable eating behaviour among employees must be reimbursed by the employer’. A mean score including the five items was computed for ‘Employer responsibility for employee health’. Internal consistency was sufficient (Cronbach’s α = 0·89).

#### Economic food environment influences

Concerning economic features three items were included, we assessed whether employees received discounts on food or drinks through work (e.g. discount on lunch orders) or a budget for lunch (e.g. employees being able to buy a free lunch up to three euros in the supermarket) which could be answered by yes/no/don’t know. When employees did receive a discount or budget, they were inquired about the specific products for which it applied, through open-ended questions. Furthermore, for each product or meal available at work, as indicated in the physical food environment section, respondents indicated whether the product or meal was for free for employees (yes/ no/ don’t know).

#### Policy food environment influences

Four items were included to determine the policy food environment. First, the presence of a food procurement policy, which is the presence of guidelines or rules to regulate the sourcing and purchasing of food products, was assessed by one item if this was present for healthy and sustainable foods (yes/no/don’t know). Second, the existence of other company food policies related to stimulating the consumption of healthy and sustainable foods (e.g. policies for collective sharing of birthday treats at work to reduce treat frequency or policies to promote a healthy eating pattern) was assessed (yes/no/don’t know). Hereafter, these policies will be referred to as ‘company food policies’. If no company food policies were present, respondents were asked to indicate whether informal agreements were present (yes/no) and if so, what they were. Third, it was assessed if (yes/no) employees were able to participate in work-supported health promotion programmes (e.g. stimulating more exercise, a healthy diet and smoking cessation). Lastly, the presence of initiatives among employees concerning policies to improve healthy and sustainable food at the workplace was assessed with a multiple-choice question where participants indicated which initiatives were present or if there were no initiatives present (Meatless Monday or other initiatives to inspire a vegetarian diet/vegan Friday or other initiatives for inspiration for a vegan diet/policies regarding treats/celebrations (at birthdays)/no-waste initiatives/no initiatives (unique option)/don’t know (unique option)/other).

### Statistical analyses

Descriptive statistics were used to describe participant socio-demographic, company and food environment characteristics. For the six items where ‘I don’t know’ was a possible answer, if a participant answered ‘I don’t know’ they were excluded from the respective analysis that involved that item. For the measures where Likert scales were used, mean values and standard deviations (SD) were calculated. These descriptive statistics were given for the total group as well as for the three independent variables (company characteristics): (1) number of employees (< 50 and ≥ 50), (2) location of work (mostly off-site *v*. on-site) and (3) availability of a worksite cafeteria (yes *v*. no).

For each dichotomised dependent food environment measure (i.e. discounts on food and drink (yes/no)), a combined binary logistic regression model was run including the three dichotomous independent variables simultaneously to evaluate their collective influence on the likelihood of the presence of the respective dependent variables. These independent variables were coded as follows: (1) number of employees (0 = < 50 employees, 1 =≥ 50 employees), (2) location of work (0 = at least half or most employees working off-site, and 1 = most employees working on-site) and (3) availability of a worksite cafeteria (0 = no worksite cafeteria, 1 = worksite cafeteria present). Multinomial logistic regression models were used similarly for the dependent measures ‘Where are lunch breaks most often spent?’ and ‘Where is lunch most often brought from?’. Odds Ratios (OR), p-values and their 95 % Confidence Intervals (CI) were presented. Linear regression models were used similarly for the dependent food environment measures which were measured on a Likert scale and of which a mean value was calculated. Regression coefficients (B) and their 95 % CI were presented. Lastly, the Pearson chi-squared and Fisher’s exact tests were used to evaluate the association between job position (employer *v*. employee) and all categorical food environment measures. The Mann–Whitney test assessed the association between job position and food environment measures where Likert scales were used. Analyses were conducted using IBM SPSS 28.0.

## Results

### Participant characteristics

Of the 315 participants, 187 (59·4 %) were male and 128 (40·6 %) were female, and the mean age was 45·6 (sd = 12·49) years. Most participants were employees of the SME (*n* 245, 77·8 %) and 22·2 % of participants were company owners (*n* 70). The participants most frequently represented the trade sector (*n* 68, 21·6 %) or business services sector (*n* 65, 20·6 %).

### Implications of COVID-19 on food and drink offer and eating practices

Changes in the offer of food and/or drinks due to COVID-19 were reported by forty-nine (15·6 %) of participants. Examples included the closure of facilities (e.g. worksite cafeteria) and less or no more available food at the workplace. Additionally, thirty-nine (12·4 %) of participants reported changes in work-related eating practices (e.g. participants eating at home, taking fewer lunch breaks together at the workplace (due to group restrictions) and a shift to healthier eating habits).

### Company characteristics

Overall, 58·4 % of SMEs had less than fifty employees (*n* 184) and 41·6 % of SMEs had fifty or more employees (*n* 131). Respondents indicated that most employees worked on-site in 67·6 % of SMEs (*n* 213) and most employees worked off-site in 32·4 % of the SMEs (*n* 102). The majority of SMEs did not have a worksite cafeteria (*n* 243, 77·1 %). Among the SMEs that had a worksite cafeteria (*n* 72), 33·3 % (*n* 24) of them had less than fifty employees, while 66·7 % (*n* 48) had fifty or more employees. Out of the SMEs that had a worksite cafeteria, most employees worked on-site (56·9 %, *n* 41) and 43·1 % (*n* 31) had most of their employees working off-site. Of the SMEs where employees worked predominantly on-site (*n* 213), 62·0 % had less than fifty employees (*n* 132) and 38·0 % had fifty or more employees (*n* 81).

### Physical food environment influences

Most SMEs did not have soft drink vending machines (*n* 219, 69·5 %), snack vending machines (*n* 250, 79·4 %), on-site fruit available (*n* 185, 58·7 %) or on-site vegetables available (*n* 230, 73·0 %) (Table 1). The majority of SMEs had a coffee and tea vending machine (*n* 281, 89·2 %) and a kitchen (*n* 247, 78·4 %). Having fifty or more employees significantly increased the likelihood that soft drink vending machines (OR = 2·71, 95 % CI 1·59, 4·61) and snack vending machines (OR = 3·93, 95 % CI = 2·08, 7·51) were present compared to having less than fifty employees, as shown in Table 2. Additionally, the likelihood of having on-site fruits and vegetables available was higher in SMEs with fifty or more employees (OR = 2·39, 95 % CI 1·42, 4·03 and OR = 1·89, 95 % CI 1·04, 3·46, respectively) than in SMEs with less than fifty employees. Having a worksite cafeteria significantly increased the likelihood of soft drink vending machines and snack vending machines being present compared to SMEs without a worksite cafeteria (OR = 4·37, 95 % CI 2·42, 7·89 and OR = 5·38, 95 % CI 2·83, 10·24, respectively). Moreover, the likelihood of having on-site fruits and vegetables available was also higher in SMEs with a worksite cafeteria (OR = 8·76, 95 % CI 4·50, 17·06 and OR = 10·29, 95 % CI 5·49, 19·31, respectively) than in SMEs without a worksite cafeteria. The location of work did not significantly increase the likelihood of any of these variables being present. No statistical differences in physical food environment influences were found based on job position (Appendix 4).

**Table 1:** Descriptive statistics, in total and stratified by company characteristics: worksite cafeteria availability, number of employees and location of work

	Total		< 50 employees		≥ 50 employees		Most employees work on-site		Most employees work off-site		With a worksite cafeteria		Without a worksite cafeteria	
	*n* 315		*n* 184		*n* 131		*n* 213		*n* 102		*n* 72		*n* 243	
	*n*	%	*n*	%	*n*	%	*n*	%	*n*	%	*n*	%	*n*	%
Physical food environment														
Soft drink vending machine														
Yes	96	30·5 %	36	19·6 %	60	45·8 %	65	30·5 %	31	30·4 %	43	59·7 %	53	21·8 %
No	219	69·5 %	148	80·4 %	71	54·2 %	148	69·5 %	71	69·6 %	29	40·3 %	190	78·2 %
Coffee and tea vending machine														
Yes	281	89·2 %	158	85·9 %	123	93·9 %	191	89·7 %	90	88·2 %	68	94·4 %	213	87·7 %
No	34	10·8 %	26	14·1 %	8	6·1 %	22	10·3 %	12	11·8 %	4	5·6 %	30	12·3 %
Snack vending machine														
Yes	65	20·6 %	18	9·8 %	47	35·9 %	45	21·1 %	20	19·6 %	35	48·6 %	30	12·3 %
No	250	79·4 %	166	90·2 %	84	64·1 %	168	78·9 %	82	80·4 %	37	51·4 %	213	87·7 %
Available fruit														
Yes	126	40·0 %	52	28·3 %	74	56·5 %	82	38·5 %	44	43·1 %	58	80·6 %	68	28·0 %
No	185	58·7 %	129	70·1 %	56	42·7 %	128	60·1 %	57	55·9 %	14	19·4 %	171	70·4 %
Don’t know	4	1·3 %	3	1·6 %	1	0·8 %	3	1·4 %	1	1·0 %	0		4	1·6 %
*If fruit is available, is it free?* *	*n* 126		*n 52*		*n 74*		*n 82*		*n 44*		*n 58*		*n* 68	
*Yes*	85	67·5 %	42	80·8 %	43	58·1 %	56	68·3 %	29	65·9 %	30	51·7 %	55	80·9 %
*No*	41	32·5 %	10	19·2 %	31	41·9 %	26	31·7 %	15	34·1 %	28	48·3 %	13	19·1 %
*Don’t know*	0		0		0		0		0		0		0	
Available vegetables														
Yes	81	25·7 %	31	16·8 %	50	38·2 %	47	22·1 %	34	33·3 %	48	66·7 %	33	13·6 %
No	230	73·0 %	151	82·1 %	79	60·3 %	164	77·0 %	66	64·7 %	24	33·3 %	206	84·8 %
Don’t know	4	1·3 %	2	1·1 %	2	1·5 %	2	0·9 %	2	2·0 %	0		4	1·6 %
*If vegetables are available, are they free?* †	*n* 81		*n 31*		*n 50*		*n 47*		*n 34*		*n 48*		*n 33*	
*Yes*	48	59·3 %	24	77·4 %	24	48·0 %	29	61·7 %	19	55·9 %	23	47·9 %	25	75·8 %
*No*	33	40·7 %	7	22·6 %	26	52·0 %	18	38·3 %	15	44·1 %	25	52·1 %	8	24·2 %
*Don’t know*	0		0		0		0		0		0		0	
Sociocultural food environment														
How are lunch breaks most often spent?														
In a common break room	192	61·0 %	100	54·3 %	92	70·2 %	138	64·8 %	54	52·9 %	53	73·6 %	139	57·2 %
Behind a desk	65	20·6 %	46	25·0 %	19	14·5 %	44	20·7 %	21	20·6 %	6	8·3 %	59	24·3 %
Out-of-the-office	58	18·4 %	38	20·7 %	20	15·3 %	31	14·5 %	27	26·5 %	13	18·1 %	45	18·5 %
Where is lunch most often brought from?														
Home	208	66·0 %	131	71·2 %	77	58·8 %	142	66·7 %	66	64·8 %	28	38·9 %	180	74·1 %
Worksite cafeteria	47	14·9 %	14	7·6 %	33	25·2 %	29	13·6 %	18	17·6 %	32	44·4 %	15	6·2 %
Purchased elsewhere	60	19·1 %	39	21·2 %	21	16·0 %	42	19·7 %	18	17·6 %	12	16·7 %	48	19·7 %
Social norms of healthy and sustainable eating at work‡														
Mean	3·28		3·25		3·31		3·31		3·20		3·44		3·23	
sd	0·56		0·54		0·59		0·56		0·57		0·52		0·57	
Employer responsibility for employee health‡														
Mean	3·18		3·09		3·30		3·19		3·16		3·42		3·10	
sd	0·76		0·75		0·75		0·74		0·79		0·75		0·75	
Economic food environment														
Discounts on food or drinks														
Yes	28	8·9 %	15	8·2 %	13	9·9 %	18	8·4 %	10	9·8 %	12	16·7 %	16	6·6 %
No	267	84·8 %	157	85·3 %	110	84·0 %	181	85·0 %	86	84·3 %	54	75·0 %	213	87·6 %
Don’t know	20	6·3 %	12	6·5 %	8	6·1 %	14	6·6 %	6	5·9 %	6	8·3 %	14	5·8 %
Budget for lunch														
Yes	15	4·8 %	7	3·8 %	8	6·1 %	9	4·2 %	6	5·9 %	5	6·9 %	10	4·1 %
No	287	91·1 %	168	91·3 %	119	90·8 %	194	91·1 %	93	91·2 %	63	87·5 %	224	92·2 %
Don’t know	13	4·1 %	9	4·9 %	4	3·1 %	10	4·7 %	3	2·9 %	4	5·6 %	9	3·7 %
Policy food environment														
Company food policy														
Yes	26	8·3 %	7	3·8 %	19	14·5 %	13	6·1 %	13	12·7 %	16	22·2 %	10	4·1 %
No	263	83·4 %	161	87·5 %	102	77·9 %	180	84·5 %	83	81·4 %	49	68·1 %	214	88·1 %
Don’t know	26	8·3 %	16	8·7 %	10	7·6 %	20	9·4 %	6	5·9 %	7	9·7 %	19	7·8 %
Health promotion programmes														
Offered	59	18·7 %	22	12·0 %	37	28·2 %	37	17·4 %	22	21·6 %	21	29·2 %	38	15·6 %
Not offered	256	81·3 %	162	88·0 %	94	71·8 %	176	82·6 %	80	78·4 %	51	70·8 %	205	84·4 %
Employee-initiated food initiatives														
Yes	55	17·5 %	17	9·2 %	38	29·0 %	28	13·2 %	27	26·5 %	23	31·9 %	32	13·2 %
No	226	71·7 %	149	81·0 %	77	58·8 %	156	73·2 %	70	68·6 %	39	54·2 %	187	76·9 %
Don’t know	34	10·8 %	18	9·8 %	16	12·2 %	29	13·6 %	5	4·9 %	10	13·9 %	24	9·9 %

* Measured by one item only shown to respondents who indicated having fruit available (*n* 126).

† Measured by one item only shown to respondents who indicated having vegetables available (*n* 81).

‡ Measured by statements indicated on a five-point Likert scale ranging from 1 ‘strongly disagree’ to 5 ‘strongly agree’.

**Table 2:** Multiple logistic regression analyses with five indicators of the physical food environment as dependent variables

	Reference category	OR	95 % CI	*P*
Soft drink vending machine				
Number of employees	≥ 50 c.t. < 50 (ref.)†	2·71	1·59, 4·61	< 0·001
Location of work	Mostly on-site c.t. Mostly off-site (ref.)	1·38	0·78, 2·45	0·27
Worksite cafeteria	Yes c.t. No (ref.)	4·37	2·42, 7·89	< 0·001
Coffee and tea vending machine				
Number of employees	≥ 50 c.t. < 50 (ref.)†	2·29	0·98, 5·36	0·06
Location of work	Mostly on-site c.t. Mostly off-site (ref.)	1·35	0·63, 2·89	0·45
Worksite cafeteria	Yes c.t. No (ref.)	1·95	0·64, 5·93	0·24
Snack vending machine				
Number of employees	≥ 50 c.t. < 50 (ref.)†	3·95	2·08, 7·51	< 0·001
Location of work	Mostly on-site c.t. Mostly off-site (ref.)	1·69	0·86, 3·32	0·13
Worksite cafeteria	Yes c.t. No (ref.)	5·38	2·83, 10·24	< 0·001
Available fruit				
Number of employees	≥ 50 c.t. < 50 (ref.)†	2·39	1·42, 4·03	0·001
Location of work	Mostly on-site c.t. Mostly off-site (ref.)	1·16	0·66, 2·03	0·60
Worksite cafeteria	Yes c.t. No (ref.)	8·76	4·50, 17·06	< 0·001
Available vegetables*				
Number of employees	≥ 50 c.t. < 50 (ref.)†	1·89	1·04, 3·46	0·04
Location of work	Mostly on-site c.t. Mostly off-site (ref.)	0·71	0·38, 1·32	0·28
Worksite cafeteria	Yes c.t. No (ref.)	10·29	5·49, 19·31	< 0·001

* Four participants were excluded from the analyses because they answered don’t know on the dependent variable.

† c.t. = compared to; (ref.) = reference category.

### Sociocultural food environment influences

In general, lunch breaks were most often spent in a common break room (*n* 192, 61·0 %) and most often brought from home (*n* 208, 66·0 %). In larger SMEs (≥ 50 employees), lunch was more often spent in a common break room and less often behind their desk (OR = 0·53, 95 % CI 0·28, 1·00) or out-of-office (OR = 0·54, 95 % CI 0·28, 1·03) than in smaller SMEs (Table 3). The likelihood of employees spending lunch out-of-the-office instead of in a common break room was significantly lower for SMEs where most employees worked on-site compared to those predominantly working off-site (OR = 0·40, 95 % CI 0·22, 0·75). Additionally, having a worksite cafeteria significantly decreased the likelihood of employees spending lunch behind a desk instead of spending it in a common break room compared to not having a worksite cafeteria (OR = 0·30, 95 % CI 0·12, 0·77). Furthermore, having fifty or more employees or having a worksite cafeteria both significantly increased the likelihood of lunch being brought from the worksite cafeteria instead of bringing lunch from home (OR = 2·39, 95 % CI 1·12, 5·11 and OR = 11·18, 95 % CI 5·24, 23·86, respectively) compared to having less than fifty employees or not having a worksite cafeteria. Employers reported that lunch breaks were significantly less often spent in a common break room and were more often spent out-of-the-office, compared to what employees reported (Appendix 4). The respondents had an average score of 3·28 (sd = 0·56, Table 1) on social norms of healthy and sustainable eating at work. Having a worksite cafeteria (B = 0·23, 95 % CI 0·08, 0·38) and working primarily on-site (B = 0·14, 95 % CI 0·01, 0·28) were significantly positively associated with stronger norms of healthy and sustainable eating at work compared to not having a worksite cafeteria and working primarily off-site, as shown in Table 4. Furthermore, the respondents had an average score of 3·18 (sd = 0·76, Table 1) on employer responsibility for employee health. Having a worksite cafeteria (B = 0·27, 95 % CI 0·07, 0·48), but not the number of employees and the location of work, was significantly positively associated with stronger perceptions that employers were responsible for employees’ health (Table 4). Employers reported significantly stronger social norms of healthy and sustainable eating at work than employees. No other statistical differences were identified in the sociocultural food environment influences based on job position (Appendix 4).

**Table 3:** Multiple multinomial logistic regression analyses with two indicators of the sociocultural food environment as dependent variables

		Lunch breaks spent behind a desk *v*. lunch spent in a common break room*			Lunch breaks spent out-of-the-office *v*. lunch spent in a common break room*		
	Reference category	OR	95 % CI	*P*	OR	95 % CI	*P*
How are lunch breaks most often spent?							
Number of employees	≥ 50 c.t. < 50 (ref.)†	0·53	0·28, 1·00	0·049	0·54	0·28, 1·03	0·06
Location of work	Mostly on-site c.t. Mostly off-site (ref.)	0·67	0·36, 1·26	0·21	0·40	0·22, 0·75	0·004
Worksite cafeteria	Yes c.t. No (ref.)	0·30	0·12, 0·77	0·01	0·79	0·38, 1·65	0·53
		Lunch brought from a worksite cafeteria *v*. lunch brought from home‡			Lunch purchased elsewhere *v*. lunch brought from home‡		
Where is lunch most often brought from?							
Number of employees	≥ 50 c.t. < 50 (ref.)†	2·39	1·12, 5·11	0·02	0·85	0·46, 1·58	0·62
Location of work	Mostly on-site c.t. Mostly off-site (ref.)	1·11	0·52, 2·35	0·80	1·12	0·60, 2·11	0·72
Worksite cafeteria	Yes c.t. No (ref.)	11·18	5·24, 23·86	< 0·001	1·70	0·79, 3·67	0·18

* Reference group = employees spent lunch in a common break room.

† c.t. = compared to; (ref.) = reference category.

‡ Reference group = employees bring lunch from home.

**Table 4:** Linear regression analyses with two combined mean scales as dependent variables; the combined mean scale from six indicators of the sociocultural food environment and the combined mean scale from five indicators of responsibility employer

		Unstandardised coefficients		Standardised coefficients			95 % CI for B
	Reference category	B	Std. error	β	t	*P*	Lower bound, upper bound
Social norms of healthy and sustainable eating at work*							
Number of employees	≥ 50 c.t. < 50 (ref.)†	0·02	0·07	0·02	0·30	0·77	−0·11, 0·15
Location of work	Mostly on-site c.t. Mostly off-site (ref.)	0·14	0·07	0·12	2·11	0·04	0·01, 0·28
Worksite cafeteria	Yes c.t. No (ref.)	0·23	0·08	0·17	2·97	0·003	0·08, 0·38
Employer responsibility for employee health*							
Number of employees	≥ 50 c.t. < 50 (ref.)†	0·16	0·09	0·11	1·81	0·07	−0·01, 0·34
Location of work	Mostly on-site c.t. Mostly off-site (ref.)	0·07	0·09	0·05	0·82	0·41	−0·10, 0·25
Worksite cafeteria	Yes c.t. No (ref.)	0·27	0·11	0·15	2·61	0·01	0·07, 0·48

* Measured by statements indicated on a five-point Likert scale ranging from 1 ‘strongly disagree’ to 5 ‘strongly agree’.

† c.t. = compared to; (ref.) = reference category.

### Economic food environment influences

In the majority of SMEs, there were no discounts on food or drinks (*n* 267, 84·8 %) or available budget for lunch at the workplace (*n* 287, 91·1 %), as shown in Table 1. Overall, 27·0 % (*n* 85) of SMEs provided free fruit whilst 15·2 % (*n* 48) of SMEs provided free vegetables. When discounts or budgets were offered, they were often not explicitly designated for specific types of products (e.g. exclusively healthy items). Only two companies explicitly stated that their discounts applied solely to healthy foods. Having a worksite cafeteria significantly increased the likelihood of discounts on food and drink being present at work compared to not having a worksite cafeteria (OR = 3·02, 95 % CI 1·29, 7·08), as shown in Table 5. Discounts were reported to be present significantly more by employers than by employees, who more often did not know whether discounts were present. No significant difference was reported in budgets available based on job position (Appendix 4).

**Table 5:** Multiple logistic regression analyses with two indicators of the economic food environment as dependent variables and three indicators of the policy food environment as dependent variables

	Reference category	OR	95 % CI	*P*
Economic food environment				
Discounts on food and drink*				
Number of employees	≥ 50 c.t. < 50 (ref.)†	0·93	0·41, 2·13	0·86
Location of work	Mostly on-site c.t. Mostly off-site (ref.)	0·99	0·43, 2·28	0·98
Worksite cafeteria	Yes c.t. No (ref.)	3·02	1·29, 7·08	0·01
Budget for lunch‡				
Number of employees	≥ 50 c.t. < 50 (ref.)†	1·42	0·48, 4·21	0·53
Location of work	Mostly on-site c.t. Mostly off-site (ref.)	0·78	0·27, 2·28	0·65
Worksite cafeteria	Yes c.t. No (ref.)	1·55	0·49, 4·95	0·46
Policy food environment				
Company food policy§				
Number of employees	≥ 50 c.t. < 50 (ref.)†	2·82	1·09, 7·29	0·03
Location of work	Mostly on-site c.t. Mostly off-site (ref.)	0·58	0·25, 1·38	0·22
Worksite cafeteria	Yes c.t. No (ref.)	5·04	2·08, 12·20	< 0·001
Health promotion programmes				
Number of employees	≥ 50 c.t. < 50 (ref.)†	2·54	1·38, 4·67	0·003
Location of work	Mostly on-site c.t. Mostly off-site (ref.)	0·89	0·48, 1·64	0·71
Worksite cafeteria	Yes c.t. No (ref.)	1·66	0·87, 3·18	0·13
Food initiatives||				
Number of employees	≥ 50 c.t. < 50 (ref.)†	3·41	1·76, 6·60	< 0·001
Location of work	Mostly on-site c.t. Mostly off-site (ref.)	0·57	0·30, 1·08	0·09
Worksite cafeteria	Yes c.t. No (ref.)	2·26	1·14, 4·46	0·02

* Twenty participants were excluded from the analyses because they answered don’t know on the dependent variable.

† c.t. = compared to; (ref.) = reference category.

‡ Thirteen participants were excluded from the analyses because they answered don’t know on the dependent variable.

§ Twenty-six participants were excluded from the analyses because they answered don’t know on the dependent variable.

|| Thirty-four participants were excluded from the analyses because they answered don’t know on the dependent variable.

### Policy food environment influences

Company food policies concerning the consumption of healthy and sustainable foods were present in a small number of SMEs (*n* 26, 8·3 %) (Table 1), and even a smaller number of SMEs (*n* 8, 2·5 %) indicated the presence of informal agreements (e.g. preferably no drinks from plastic bottles or a joint birthday treat by the SME once a month). Furthermore, the minority of SMEs with a worksite cafeteria had food procurement policies (*n* 14, 19·4 %). Most SMEs did not offer health promotion programmes (*n* 256, 81·3 %) nor employee-initiated food initiatives (*n* 226, 71·7 %). The most prevalent food initiatives that were present were initiatives regarding treats/celebrations or no-waste initiatives (both *n* 20, 6·3 %). Having fifty or more employees significantly increased the likelihood of the presence of company food policies (OR = 2·82, 95 % CI 1·09, 7·29), health promotion programmes (OR = 2·54, 95 % CI 1·38, 4·67) and food initiatives (OR = 3·41, 95 % CI 1·76, 6·60) (Table 5) compared to having less than fifty employees. In addition, the likelihood of the presence of company food policies (OR = 5·04, 95 % CI 2·08, 12·20) and food initiatives (OR = 2·26, 95 % CI 1·14, 4·46) was significantly higher when a worksite cafeteria was present than when no worksite cafeteria was present. Company food policies were reported to be present significantly more by employers than by employees, who more often did not know whether policies were present. No significant difference was reported in health promotion programmes and employee-initiated food initiatives based on job position (Appendix 4).

## Discussion

This study showed that the majority of SMEs did not have a worksite cafeteria and most SMEs did not have facilities such as vending machines nor offered lunch, fruit and vegetables. At SMEs, lunch was often brought from home and lunch breaks were most often spent in a common break room. Most SMEs neither offered lunch discounts nor provided a lunch budget to their employees. Furthermore, most SMEs lacked health promotion programmes, company food policies and employee-initiated food initiatives. Additionally, company characteristics were significantly associated with food environment influences of SMEs.

Our findings show a stronger social norm of healthy and sustainable eating at work when employees work on-site and a worksite cafeteria is present compared to when no worksite cafeteria is present and employees work mostly off-site. As suggested by Escoffery and colleagues, this can be attributed to the dependency on external food outlets in proximities of SMEs (e.g. supermarkets and bakeries) or on-the-go options (e.g. petrol stations and restaurants), if employees do not have a worksite cafeteria or work off-site and do not bring their lunch from home^(28)^. Such outlets predominantly offer less healthy food options^(45)^, and the reduced proximity to healthy food options can cause employees during working hours, to perceive unhealthy food consumption as common and appropriate^(41)^ and thus shape unhealthier social norms^(41)^.

Our study showed that SMEs with fewer employees were less likely to have health promotion programmes, company food policies and employee-initiated food initiatives compared to their larger counterparts. This is in line with a systematic review by McCoy et al.^(29)^, finding that fewer small businesses adopt health promotion programmes compared to larger businesses. Small businesses may face barriers such as costs, lack of employee interest, a lack of management support and expertise that hinder the implementation of health promotion programmes. Also, managers’ fear of ‘paternalistic’ image and avoiding stigmatising individuals have been observed as reasons that hindered the implementation of health promotion programmes in smaller businesses^(29)^. These findings may indicate that healthy lifestyles (e.g. eating behaviours) are more often supported by larger than smaller SMEs. However, in our study, we observed that *required* employer responsibility for employee health was similar among all SME sizes. This might raise the issue of environmental injustice at the work floor, which defines the lack of equal access to a healthy environment in which to live, learn and work^(46)^. This is further accentuated by the evident disparity in other health-promoting features of the workplace food environment (e.g. the lack of availability of fruits and vegetables).

From the results, it is notable that the location of work only impacted features of the sociocultural food environment (social norms and where lunch was spent), whereas no considerable differences in the physical, economic or policy food environment were observed between SMEs where employees primarily worked on-site *v*. off-site. Based on findings of Seward et al.^(32)^, that showed that employees working off-site generally have access to fewer health promotion programmes compared to those working on-site, it could be hypothesised that on-site work environments prioritise employee health more substantially than off-site work environments and would therefore have more policies and initiatives available. However, we did not confirm this hypothesis with this study. It should however be noted that the adoption of food environment supportive policies or economic incentives was relatively low in the entire included sample of SMEs.

Our study highlighted the different work food environment perceptions between employers and employees. Employers were found to be better aware of the available workplace amenities, as indicated by their higher reported availability of company food policies and discounts compared to employees. However, employers rated stronger social norms in favour of healthy and sustainable eating at work compared to employees and thought employees spent lunch breaks more frequently outside. This may suggest that employers are not necessarily aware of the practices at the actual work floor. Yet, it should be acknowledged that employers and employees participating in this study do not all represent the same company, and the majority of participants comprised employees. Nevertheless, these observations require additional understanding of the gap between employers and employees, not just in terms of awareness of organisational policies but also in the day-to-day dynamics that shape the workplace experience.

Whilst conducting our research, Castro and colleagues^(14)^ developed a more specific model for the organisational food environment. This model does reflect that the infrastructure of an organisation can allow employees to take food from home and thus shape the food environment when no food is commercially offered, which is a valuable addition for SMEs. However, the sociocultural food environment as observed in our study, where social norms of healthy and sustainable eating also play a role in the SME food environment, is currently not integrated in this model by Castro. Therefore, our findings contribute to expanding Castro’s conceptualisation of the organisational food environment by proposing the inclusion of the sociocultural food environment in this model.

Since the survey was conducted during the COVID-19 pandemic in the Netherlands, the consequential working from home may have influenced the workplace food environment and eating practices. Although the results show that only a minority (about 16 %) of the participants reported changes in eating facilities and practices at work, it is essential to acknowledge this when interpreting the results. Moreover, it is arguable that trends such as increased remote work may have lasting effects on the workplace food environment^(47)^. However, to gain a deeper understanding, further research is needed to explore the lasting impact of COVID-19 and remote work on the workplace food environment.

While this study has noteworthy strengths, such as its relatively large study sample and variety of businesses included, it is not without its limitations. Given the cross-sectional nature of the data, no causal relationships can be identified. All outcome measures were self-reported which may have caused recall bias. However, given that the study primarily revolved around the presence of items such as policies and snack machines, rather than behavioural aspects, the data should remain reliable. Finally, in the study, we recruited ‘employees and employers of SMEs who were (co)responsible for the ‘food and drink’ within their company. This may have resulted in an underrepresentation of SMEs that offer no food or drinks at all. However, since most companies have coffee and tea available, participants solely responsible for coffee procurement were included, and coffee services are commonly provided in the Netherlands, even if food policy or catering is absent^(48)^.

Future research should focus on which existing strategies in the food environments in Dutch SMEs are most effective in addressing eating practices and feasible to implement in the different SME company types. Additionally, future research should explore the lasting impact of COVID-19 and remote work on the workplace food environment.

### Conclusion

Given the fact that more than 99 % of all companies in the Netherlands and the EU are classified as SMEs, creating food environments in SMEs that stimulate healthy and sustainable eating behaviour is crucial. In SMEs, an overall comprehensive picture of the food environment points to its limited active encouragement of healthy food choices, particularly so in small SMEs without a worksite cafeteria. Additionally, the location of work was only found to influence features of the sociocultural food environment and made no significant difference to the physical, economic or policy food environment. Therefore, future research should focus on which existing strategies in the food environments in Dutch SMEs are most effective in addressing eating practices and feasible to implement in the different SME company types.

## Supporting information

Geboers et al. supplementary material Geboers et al. supplementary material

## Supplementary material

For supplementary material accompanying this paper visit https://doi.org/10.1017/S1368980024000946
