# Supplementary material for: More than the worksite cafeteria: the workplace food environment of small and medium-sized enterprises in the Netherlands
Source: Public Health Nutr. 2024 Apr 29;27(1):e137. doi: 10.1017/S1368980024000946 (PMC11374566; doi:10.1017/S1368980024000946)
Supplement: Geboers et al. supplementary material [file S1368980024000946sup001.docx]

**Appendix 1**

**Table 1** Kitchen appliances in total and stratified by: worksite cafeteria availability, number of employees, and location of work

|  | Total | <50  employees | ≥50  employees | Most  employees work on-site | Most employees work off-site | With a worksite cafeteria | Without a worksite cafeteria |
| --- | --- | --- | --- | --- | --- | --- | --- |
|  | (*n* 247)* | (*n* 148) | (*n* 99) | (*n* 170) | (*n* 77) | (*n* 52) | (*n* 195) |
|  | *n* and % | *n* and % | *n* and % | *n* and % | *n* and % | *n* and % | *n* and % |
| **Microwave**  Yes  No | 201 (81·4%)  46 (18·6%) | 115 (77·7%)  33 (22·3%) | 86 (86·9%)  13 (13·1%) | 134 (78·8%)  36 (21·2%) | 67 (87·0%)  10 (13·0%) | 46 (88·5%)  6 (11·5%) | 155 (79·5%)  40 (20·5%) |
| **Frying pan**  Yes  No | 33 (13·4%)  214 (86·6%) | 18 (12·2%)  130 (87·8%) | 15 (15·2%)  84 (84·8%) | 19 (11·2%)  151 (88·8%) | 14 (18·2%)  63 (81·8%) | 13 (25·0%)  39 (75·0%) | 20 (10·3%)  175 (89·7%) |
| **Airfryer**  Yes  No | 30 (12·1%)  217 (87·9%) | 18 (12·2%)  130 (87·8%) | 12 (12·1%)  87 (87·9%) | 20 (11·8%)  150 (88·2%) | 10 (13·0%)  67 (87·0%) | 14 (26·9%)  38 (73·1%) | 16 (8·2%)  179 (91·8%) |
| **Panini grill/ electric grill**  Yes  No | 133 (53·8%)  114 (46·2%) | 82 (55·4%)  66 (44·6%) | 51 (51·5%)  48 (48·5%) | 89 (52·4%)  81 (47·6%) | 44 (57·1%)  33 (42·9%) | 34 (65·4%)  18 (34·6%) | 99 (50·8%)  96 (49·2%) |
| **Toaster**  Yes  No | 57 (23·1%)  190 (76·9%) | 35 (23·6%)  113 (76·4%) | 22 (22·2%)  77 (77·8%) | 39 (22·9%)  131 (77·1%) | 18 (23·4%)  59 (76·6%) | 17 (32·7%)  35 (67·3%) | 40 (20·5%)  155 (79·5%) |
| **Kettle**  Yes  No | 172 (69·6%)  75 (30·4%) | 113 (76·4%)  35 (23·6%) | 59 (59·6%)  40 (40·4%) | 114 (67·1%)  56 (32·9%) | 58 (75·3%)  19 (24·7%) | 33 (63·5%)  19 (36·5%) | 139 (71·3%)  56 (28·7%) |
| **Coffee-machine**  Yes  No | 188 (76·1%)  59 (23·9%) | 118 (79·7%)  30 (20·3%) | 70 (70·7%)  29 (29·3%) | 119 (70·0%)  51 (30·0%) | 69 (89·6%)  8 (10·4%) | 36 (69·2%)  16 (30·8%) | 152 (77·9%)  43 (22·1%) |
| **Refrigerator**  Yes  No | 235 (95·1%)  12 (4·9%) | 145 (98·0%)  3 (2·0%) | 90 (90·9%)  9 (9·1%) | 160 (94·1%)  10 (5·9%) | 75 (97·4%)  2 (2·6%) | 49 (94·2%)  3 (5·8%) | 186 (95·4%)  9 (4·6%) |
| **Freezer**  Yes  No | 118 (47·8%)  129 (52·2%) | 71 (48·0%)  77 (52·0%) | 47 (47·5%)  52 (52·5%) | 74 (43·5%)  96 (56·5%) | 44 (57·1%)  33 (42·9%) | 29 (55·8%)  23 (44·2%) | 89 (45·6%)  106 (54·4%) |
| **Blender**  Yes  No | 15 (6·1%)  232 (93·9%) | 9 (6·1%)  139 (93·9%) | 6 (6·1%)  93 (93·9%) | 9 (5·3%)  161 (94·7%) | 6 (7·8%)  71 (92·2%) | 8 (15·4%)  44 (84·6%) | 7 (3·6%)  188 (96·4%) |
| **Tableware (cutlery, plates, etc.)**  Yes  No | 188 (76·1%)  59 (23·9%) | 122 (82·4%)  26 (17·6%) | 66 (66·7%)  33 (33·3%) | 129 (75·9%)  41 (24·1%) | 59 (76·6%)  18 (23·4%) | 44 (84·6%)  8 (15·4%) | 144 (73·8%)  51 (26·2%) |

* Measured only by respondents who indicated having vegetables available (*n* 81)

**Appendix 2**

**Table 2** Availability and cost of food and drink products in total and stratified by: worksite cafeteria, number of employees and location of work

|  | Total | <50  employees | ≥50  employees | Most  employees work on-site | Most employees work off-site | With a worksite cafeteria | Without a worksite cafeteria |
| --- | --- | --- | --- | --- | --- | --- | --- |
|  | (*n* 121)* | (*n* 60) | (*n* 61) | (*n* 77) | (*n* 44) | (*n* 72) | (*n* 49) |
|  | *n* and % | *n* and % | *n* and % | *n* and % | *n* and % | *n* and % | *n* and % |
| **Nuts**  Yes  No  Don’t know | 29 (24·0%)  91 (75·2%)  1 (0·8%) | 7 (11·7%)  53 (88·3%)  0 | 22 (36·1%)  38 (62·3%)  1 (1·6%) | 14 (18·2%)  62 (80·5%)  1 (1·3%) | 15 (34·1%)  29 (65·9%)  0 | 22 (30·6%)  49 (68·1%)  1 (1·3%) | 7 (14·3%)  42 (85·7%)  0 |
| **If nuts are available, are they free?†**  Yes  No  Don’t know | *n* 29  18 (62·1%)  11 (37·9%)  0 | *n* 7  6 (85·7%)  1 (14·3%)  0 | *n* 22  12 (54·5%)  10 (45·5%)  0 | *n* 14  9 (64·3%)  5 (35·7%)  0 | *n* 15  9 (60·0%)  6 (40·0%)  0 | *n* 22  13 (59·1%)  9 (40·9%)  0 | *n* 7  5 (71·4%)  2 (28·6%)  0 |
| **Sweet snacks**  Yes  No | 77 (63·6%)  44 (36·4%) | 32 (53·3%)  28 (46·7%) | 45 (73·8%)  16 (26·2%) | 47 (61·0%)  30 (39·0%) | 30 (68·2%)  14 (31·8%) | 52 (72·2%)  20 (27·8%) | 25 (51·0%)  24 (49·0%) |
| **If sweet snacks are available, are they free?†**  Yes  No  Don’t know | *n* 77  37 (48·1%)  39 (50·6%)  1 (1·3%) | *n* 32  22 (68·8%)  9 (28·1%)  1 (3·1%) | *n* 45  15 (33·3%)  30 (66·7%)  0 | *n* 47  20 (42·6%)  26 (55·3%)  1 (2·1%) | *n* 30  17 (56·7%)  13 (43·3%)  0 | *n* 52  19 (36·5%)  32 (61·5%)  1 (2·0%) | *n* 25  18 (72·0%)  7 (28·0%)  0 |
| **Savoury snacks**  Yes  No | 50 (41·3%)  71 (58·7%) | 19 (31·7%)  41 (68·3%) | 31 (50·8%)  30 (49·2%) | 27 (35·1%)  50 (64·9%) | 23 (52·3%)  21 (47·7%) | 34 (47·2%)  38 (52·8%) | 16 (32·7%)  33 (67·3%) |
| **If savoury snacks are available, are they free**?†  Yes  No  Don’t know | *n* 50  22 (44·0%)  27 (54·0%)  1 (2·0%) | *n* 19  12 (63·2%)  6 (31·6%)  1 (5·3%) | *n* 31  10 (32·3%)  21 (67·7%)  0 | *n* 27  7 (25·9%)  19 (70·4%)  1 (3·7%) | *n* 23  15 (65·2%)  8 (34·8%)  0 | *n* 34  12 (35·3%)  21 (61·8%)  1 (2·9%) | *n* 16  10 (62·5%)  6 (37·5%)  0 |
| **Hot fried snacks**  Yes  No | 44 (36·4%)  77 (63·6%) | 20 (33·3%)  40 (66·7%) | 24 (39·3%)  37 (60·7%) | 25 (32·5%)  52 (67·5%) | 19 (43·2%)  25 (56·8%) | 34 (47·2%)  38 (52·8%) | 10 (20·4%)  39 (79·6%) |
| **If hot fried snacks are available, are they free?†**  Yes  No  Don’t know | *n* 44  19 (43·2%)  24 (54·5%)  1 (2·3%) | *n* 20  11 (55·0%)  8 (40·0%)  1 (5·0%) | *n* 24  8 (33·3%)  16 (66·7%)  0 | *n* 25  11 (44·0%)  13 (52·0%)  1 (4·0%) | *n* 19  8 (42·1%)  11 (57·9%)  0 | *n* 34  9 (26·5%)  24 (70·6%)  1 (2·9%) | *n* 10  10 (100·0%)  0  0 |
| **(butter) Milk**  Yes  No  Don’t know | 76 (62·8%)  44 (36·4%)  1 (0·8%) | 29 (48·3%)  31 (51·7%)  0 | 47 (77·1%)  13 (21·3%)  1 (1·6%) | 45 (58·4%)  31 (40·3%)  1 (1·3%) | 31 (70·5%)  13 (29·5%)  0 | 57 (79·2%)  15 (20·8%)  0 | 19 (38·8%)  29 (59·2%)  1 (2·0%) |
| **If (butter)milk is available, is it free?†**  Yes  No  Don’t know | *n* 76  37 (48·7%)  39 (51·3%)  0 | *n* 29  18 (62·1%)  11 (37·9%)  0 | *n* 47  19 (40·4%)  28 (59·6%)  0 | *n* 45  21 (46·7%)  24 (53·3%)  0 | *n* 31  16 (51·6%)  15 (48·4%)  0 | *n* 57  24 (42·1%)  33 (57·9%)  0 | *n* 19  13 (68·4%)  6 (31·6%)  0 |
| **Plant-based drinks**  Yes  No  Don’t know | 38 (31·4%)  81 (66·9%)  2 (1·7%) | 9 (15·0%)  51 (85·0%)  0 | 29 (47·5%)  30 (49·2%)  2 (3·3%) | 21 (27·3%)  55 (71·4%)  1 (1·3%) | 17 (38·6%)  26 (59·1%)  1 (2·3%) | 32 (44·4%)  38 (52·8%)  2 (2·8%) | 6 (12·2%)  43 (87·8%)  0 |
| **If plant-based drinks are available, are they free?†**  Yes  No  Don’t know | *n* 38  17 (44·7%)  21 (55·3%)  0 | *n* 9  6 (66·7%)  3 (33·3%)  0 | *n* 29  11 (37·9%)  18 (62·1%)  0 | *n* 21  9 (42·9%)  12 (57·1%)  0 | *n* 17  8 (47·1%)  9 (52·9%)  0 | *n* 32  14 (43·7%)  18 (56·3%)  0 | *n* 6  3 (50·0%)  3 (50·0%)  0 |
| **Sugary soft drinks**  Yes  No  Don’t know | 91 (75·2%)  27 (22·3%)  3 (2·5%) | 40 (66·7%)  19 (31·7%)  1 (1·6%) | 51 (83·6%)  8 (13·1%)  2 (3·3%) | 54 (70·1%)  20 (26·0%)  3 (3·9%) | 37 (84·1%)  7 (15·9%)  0 | 62 (86·1%)  9 (12·5%)  1 (1·4%) | 29 (59·2%)  18 (36·7%)  2 (4·1%) |
| **If sugary soft drinks are available, are they free?†**  Yes  No  Don’t know | *n* 91  45 (49·5%)  46 (50·5%)  0 | *n* 40  27 (67·5%)  13 (32·5%)  0 | *n* 51  18 (35·3%)  33 (64·7%)  0 | *n* 54  24 (44·4%)  30 (55·6%)  0 | *n* 37  21 (56·8%)  16 (43·2%)  0 | *n* 62  23 (37·1%)  39 (62·9%)  0 | *n* 29  22 (75·9%)  7 (24·1%)  0 |
| **Soft drinks with less or no sugar**  Yes  No  Don’t know | 89 (73·5%)  29 (24·0%)  3 (2·5%) | 39 (65·0%)  20 (33·3%)  1 (1·7%) | 50 (82·0%)  9 (14·7%)  2 (3·3%) | 49 (63·6%)  25 (32·5%)  3 (3·9%) | 40 (90·9%)  4 (9·1%)  0 | 61 (84·7%)  9 (12·5%)  2 (2·8%) | 28 (57·2%)  20 (40·8%)  1 (2·0%) |
| **If soft drinks with less/no sugar are available, are they free?†**  Yes  No  Don’t know | *n* 89  44 (49·4%)  44 (49·4%)  1 (1·2%) | *n* 39  28 (71·8%)  11 (28·2%)  0 | *n* 50  16 (32·0%)  33 (66·0%)  1 (2·0%) | *n* 49  22 (44·9%)  26 (53·1%)  1 (2·0%) | *n* 40  22 (55·0%)  18 (45·0%)  0 | *n* 61  22 (36·1%)  38 (62·3%)  1 (1·6%) | *n* 28  22 (78·6%)  6 (21·4%)  0 |
| **Preprepared sandwich**  Yes  No | 57 (47·1%)  64 (52·9%) | 17 (28·3%)  43 (71·7%) | 40 (65·6%)  21 (34·4%) | 32 (41·6%)  45 (58·4%) | 25 (56·8%)  19 (43·2%) | 50 (69·4%)  22 (30·6%) | 7 (14·3%)  42 (85·7%) |
| **If pre-prepared sandwiches are available, are they free?†**  Yes  No  Don’t know | *n* 57  15 (26·3%)  42 (73·7%)  0 | *n* 17  6 (35·3%)  11 (64·7%)  0 | *n* 40  9 (22·5%)  31 (77·5%)  0 | *n* 32  9 (28·1%)  23 (71·9%)  0 | *n* 25  6 (24·0%)  19 (76·0%)  0 | *n* 50  10 (20·0%)  40 (80·0%)  0 | *n* 7  5 (71·4%)  2 (28·6%)  0 |
| **Bread slices and/or crackers**  Yes  No | 59 (48·8%)  62 (51·2%) | 21 (35·0%)  39 (65·0%) | 38 (62·3%)  23 (37·7%) | 29 (37·7%)  48 (62·3%) | 30 (68·2%)  14 (31·8%) | 47 (65·3%)  25 (34·7%) | 12 (24·5%)  37 (75·5%) |
| **If bread slices and/or crackers are available, are they free?†**  Yes  No  Don’t know | *n* 59  21 (35·6%)  38 (64·4%)  0 | *n* 21  10 (47·6%)  11 (52·4%)  0 | *n* 38  11 (28·9%)  27 (71·1%)  0 | *n* 29  8 (27·6%)  21 (72·4%)  0 | *n* 30  13 (43·3%)  17 (56·7%)  0 | *n* 47  12 (25·5%)  35 (74·5%)  0 | *n* 12  9 (75·0%)  3 (25·0%)  0 |
| **Sweet or savoury pastries (e.g., croissants, sausage rolls, cinnamon rolls)**  Yes  No  Don’t know | 46 (38·0%)  74 (61·2%)  1 (0·8%) | 13 (21·7%)  47 (78·3%)  0 | 33 (54·1%)  27 (44·3%)  1 (1·6%) | 26 (33·8%)  50 (64·9%)  1 (1·3%) | 20 (45·5%)  24 (54·5%)  0 | 39 (54·2%)  33 (45·8%)  0 | 7 (14·3%)  41 (83·7%)  1 (2·0%) |
| **If sweet or savoury pastries are available, are they free?†**  Yes  No  Don’t know | *n* 46  13 (28·3%)  33 (71·7%)  0 | *n* 13  4 (30·8%)  9 (69·2%)  0 | *n* 33  9 (27·3%)  24 (72·7%)  0 | *n* 26  7 (26·9%)  19 (73·1%)  0 | *n* 20  6 (30·0%)  14 (70·0%)  0 | *n* 39  8 (20·5%)  31 (79·5%)  0 | *n* 7  5 (71·4%)  2 (28·6%)  0 |
| **Sweet bread toppings (e.g., 1 box of sprinkles)**  Yes  No  Don’t know | 59 (48·8%)  61 (50·4%)  1 (0·8%) | 22 (36·7%)  38 (63·3%)  0 | 37 (60·7%)  23 (37·7%)  1 (1·6%) | 29 (37·7%)  47 (61·0%)  1 (1·3%) | 30 (68·2%)  14 (31·8%)  0 | 46 (63·9%)  26 (36·1%)  0 | 13 (26·5%)  35 (71·5%)  1 (2·0%) |
| **If sweet bread toppings are available, are they free?†**  Yes  No  Don’t know | *n* 59  23 (39·0%)  35 (59·3%)  1 (1·7%) | *n* 22  11 (50·0%)  10 (45·5%)  1 (4·5%) | *n* 37  12 (32·4%)  25 (67·6%)  0 | *n* 29  12 (41·4%)  16 (55·2%)  1 (3·4%) | *n* 30  11 (36·7%)  19 (63·3%)  0 | *n* 46  13 (28·2%)  32 (69·6%)  1 (2·2%) | *n* 13  10 (76·9%)  3 (23·1%)  0 |
| **Cheese or eggs for on bread and/or crackers(e.g., 1 slice of cheese, egg)**  Yes  No  Don’t know | 69 (57·0%)  51 (42·2%)  1 (0·8%) | 25 (41·7%)  35 (58·3%)  0 | 44 (72·2%)  16 (26·2%)  1 (1·6%) | 42 (54·5%)  34 (44·2%)  1 (1·3%) | 27 (61·4%)  17 (38·6%)  0 | 53 (73·6%)  19 (26·4%)  0 | 16 (32·7%)  32 (65·3%)  1 (2·0%) |
| **If cheese or eggs are available, are they free?†**  Yes  No  Don’t know | *n* 69  27 (39·1%)  42 (60·9%)  0 | *n* 25  13 (52·0%)  12 (48·0%)  0 | *n* 44  14 (31·8%)  30 (68·2%)  0 | *n* 42  18 (42·9%)  24 (57·1%)  0 | *n* 27  9 (33·3%)  18 (66·7%)  0 | *n* 53  15 (28·3%)  38 (71·7%)  0 | *n* 16  12 (75·0%)  4 (25·0%)  0 |
| **Cold cuts for on bread and/or crackers (e.g., 1 slice of ham)**  Yes  No | 72 (59·5%)  49 (40·5%) | 26 (43·3%)  34 (56·7%) | 46 (75·4%)  15 (24·6%) | 42 (54·5%)  35 (45·5%) | 30 (68·2%)  14 (31·8%) | 55 (76·4%)  17 (23·6%) | 17 (34·7%)  32 (65·3%) |
| **If cold cuts are available, are they free?†**  Yes  No  Don’t know | *n* 72  30 (41·7%)  42 (58·3%)  0 | *n* 26  14 (53·8%)  12 (46·2%)  0 | *n* 46  16 (34·8%)  30 (65·2%)  0 | *n* 42  17 (40·5%)  25 (59·5%)  0 | *n* 30  13 (43·3%)  17 (56·7%)  0 | *n* 55  17 (30·9%)  38 (69·1%)  0 | *n* 17  13 (76·5%)  4 (23·5%)  0 |
| **Plant-based spreads for on bread and/or crackers (e.g., grilled vegetables, hummus)**  Yes  No  Don’t know | 36 (29·8%)  84 (69·4%)  1 (0·8%) | 10 (16·7%)  49 (81·7%)  1 (1·6%) | 26 (42·6%)  35 (57·4%)  0 | 19 (24·7%)  58 (75·3%)  0 | 17 (38·6%)  26 (59·1%)  1 (2·3%) | 30 (41·7%)  41 (56·9%)  1 (1·4%) | 6 (12·2%)  43 (87·8%)  0 |
| **If plant-based spreads are available, are they free?†**  Yes  No  Don’t know | *n* 36  16 (44·4%)  20 (55·6%)  0 | *n* 10  5 (50·0%)  5 (50·0%)  0 | *n* 26  11 (42·3%)  15 (57·7%)  0 | *n* 19  9 (47·4%)  10 (52·6%)  0 | *n* 17  7 (41·2%)  10 (58·8%)  0 | *n* 30  12 (40·0%)  18 (60·0%)  0 | *n* 6  4 (66·7%)  2 (33·3%)  0 |
| **Soups (excluding instant soups such as 'Cup a Soup')**  Yes  No | 46 (38·0%)  75 (62·0%) | 15 (25·0%)  45 (75·0%) | 31 (50·8%)  30 (49·2%) | 29 (37·7%)  48 (62·3%) | 17 (38·6%)  27 (61·4%) | 38 (52·8%)  34 (47·2%) | 8 (16·3%)  41 (83·7%) |

* The total group is only comprised of respondents who indicated having other products and/or meals available at work besides fruits and vegetables

† Measured only by respondents who indicated that the product was available

**Appendix 3**

**Table 3** Explanation of concepts healthy diet and sustainable diet

| Concept | Explanation |
| --- | --- |
| Healthy diet | A diet including fruit, salads or other vegetables, unsalted nuts, whole-grain cereal products such as whole-wheat bread, 30+ cheese, low-fat or semi-skimmed dairy products, water, tea and few sweets, snacks and sugary drinks. |
| Sustainable diet | A diet including little meat, few dairy products such as milk, yoghurt and cheese and more vegetable or vegetarian products such as legumes, nuts and milk substitutes such as soy milk and oat milk. Additionally, a sustainable diet can consider combating food waste and reducing the portions of meat and dairy, such as taking 1 slice of cheese instead of 2 slices of cheese. |

**Appendix 4**

**Table 4** Descriptive statistics, in total and stratified by job position (employers vs. employee)

| Characteristics | Total | Employers | Employees | *P* |
| --- | --- | --- | --- | --- |
|  | *n* 315 | *n* 70 | *n* 245 |  |
|  | *n* and % | *n* and % | *n* and % |  |
| **Physical food environment** | | | | |
| **Soft drink vending machine**  Yes  No | 96 (30·5%)  219 (69·5%) | 23 (32·9%)  47 (67·1%) | 73 (29·8%)  172 (70·2%) | 0·62 |
| **Coffee-and tea vending machine**  Yes  No | 281 (89·2%)  34 (10·8%) | 59 (84·3%)  11 (15·7%) | 222 (90·6%)  23 (9·4%) | 0·13 |
| **Snack vending machine**  Yes  No | 65 (20·6%)  250 (79·4%) | 11 (15·7%)  59 (84·3%) | 54 (22·0%)  191 (78·0%) | 0·25 |
| **Available fruit**  Yes  No  Don’t know | 126 (40·0%)  185 (58·7%)  4 (1·3%) | 35 (50·0%)  34 (84·6%)  1 (1·4%) | 91 (37·2%)  151 (61·6%)  3 (1·2%) | 0·11 |
| ***If fruit is available, is it free?****  *Yes*  *No*  *Don’t know* | *n* 126  85 (67·5%)  41 (32·5%)  0 | *n* 35  22 (62·9%)  13 (37·1%)  0 | *n* 91  63 (69·2%)  28 (30·8%)  0 | 0·49 |
| **Available vegetables**  Yes  No  Don’t know | 81 (25·7%)  230 (73·0%)  4 (1·3%) | 20 (28·6%)  49 (70·0%)  1 (1·4%) | 61 (24·9%)  181 (73·9%)  3 (1·2%) | 0·68 |
| ***If vegetables are available, are they free?*†**  *Yes*  *No*  *Don’t know* | *n* 81  48 (59·3%)  33 (40·7%)  0 | *n* 20  11 (55·*0%)*  9 (45·0%)  0 | *n* 61  37 (60·7%)  24 (39·3%)  0 | 0·66 |
| **Sociocultural food environment** | | | | |
| **How are lunch breaks most often spent?**  In a common break room  Behind a desk  Out of the office | 192 (61·0%)  65 (20·6%)  58 (18·4%) | 33 (47·1%)_a_  13 (18·6%)_a_  24 (34·3%)_a_ | 159 (64·9%)_b_  52 (21·2%)_a_  34 (13·9%)_b_ | **<0·001** |
| **Where is lunch most often brought from?**  Home  Worksite cafeteria  Purchased elsewhere | 208 (66·0%)  47 (14·9%)  60 (19·1%) | 42 (60·0%)  13 (18·6%)  15 (21·4%) | 166 (67·7%)  34 (13·9%)  45 (18·4%) | 0·46 |
| **Social norms of healthy and sustainable eating at work**‡,  mean and SD | 3·28 (0·56) | 3·45 (0·49) | 3·23 (0·57) | **0·01** |
| **Employer responsibility for employee health**‡, mean and SD | 3·18 (0·76) | 3·15 (0·84) | 3·19 (0·73) | 0·46 |
| **Economic food environment** | | | | |
| **Discounts on food or drinks**  Yes  No  Don’t know | 28 (8·9%)  267 (84·8%)  20 (6·3%) | 11 (15·7%)_a_  59 (84·3%)_a_  0_a_ | 17 (6·9%)_b_  208 (84·9%)_a_  20 (8·2%)_b_ | **0·003** |
| **Budget for lunch**  Yes  No  Don’t know | 15 (4·8%)  287 (91·1%)  13 (4·1%) | 4 (5·7%)  65 (92·9%)  1 (1·4%) | 11 (4·5%)  222 (90·6%)  12 (4·9%) | 0·44 |
| **Policy food environment** | | | | |
| **Company food policy**  Yes  No  Don’t know | 26 (8·3%)  263 (83·4%)  26 (8·3%) | 11 (15·7%)_a_  55 (78·6%)_a_  4 (5·7%)_a_ | 15 (6·1%)_b_  208 (84·9%)_a_  22 (9·0%)_a_ | **0·03** |
| **Health promotion programmes**  Offered  Not offered | 59 (18·7%)  256 (81·3%) | 18 (25·7%)  52 (74·3%) | 41 (16·7%)  204 (83·3%) | 0·09 |
| **Employee-initiated food initiatives**  Yes  No  Don’t know | 55 (17·5%)  226 (71·7%)  34 (10·8%) | 15 (21·4%)  49 (70·0%)  6 (8·6%) | 40 (16·3%)  177 (72·2%)  28 (11·4%) | 0·53 |

P-values indicate if a statistically significant difference exists between employers and employees, using Mann-Whitney tests for continuous variables and Pearson chi-squared test and Fisher's exact test for categorical variables, at a 95 % CI.

* Measured by one item only shown to respondents who indicated having fruit available (*n* 126)

† Measured by one item only shown to respondents who indicated having vegetables available (*n* 81)

‡ Measured by statements indicated on a five-point Likert scale ranging from 1 ‘strongly disagree’ to 5 ‘strongly agree’
